# Optimizing oncolytic virus design: a “Swiss army knife” approach to create a systemically delivered therapeutic

**DOI:** 10.1038/s41392-024-01815-2

**Published:** 2024-04-02

**Authors:** Carolina S. Ilkow, John Cameron Bell

**Affiliations:** Ottawa Hospital Research Institute, 501 Smyth Road, Ottawa, ON K1H 8L6 Canada

**Keywords:** Drug development, Drug development

In a recent paper published in *Nature Biotechnology*, Chen et al.^1^ describe a multiplex oncolytic virus platform that coupled with adoptive T cell therapy could solve the problem of repeat, systemic delivery of virus based therapeutics.

**Fig. 1:**
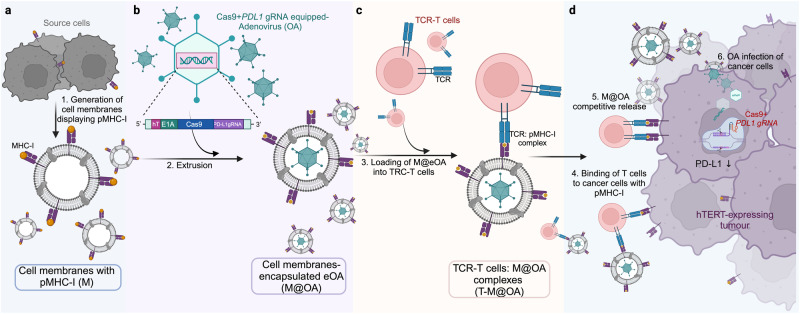
T Cell Mediated Delivery of Cloaked Virus Particles. Multi-pronged approach to oncolytic adenovirus engineering by Chen et al. **a** Cell membranes or vesicles are generated from cancer cells or cells engineered to display T-cell specific tumor antigens [e.g., peptide: MHC-I complex (pMHC-I]; **b** An engineered oncolytic adenovirus (OA) with E1A gene under hTERT promoter (hT) control and encoding Cas9 enzyme and guide RNAs against PDL1 is encapsulated into cell membranes displaying pMHC-I (M@OA). **c** Co-incubation of M@OA with TRC-T cells leads to immobilization of M@OA onto carrier T cells (T-M@OA). **d** OA released from carrier T cells targets tumor cells via specific antigens, leading to virus internalization and infection. Expression of Cas9 enzyme and PDL1 gRNAs from the OA backbone disrupts the PDL1 gene, reducing immunosuppression and enhancing T cell-mediated cancer cell killing. Created with Biorender

It is widely accepted that multi-targeted therapeutic approaches attacking several facets of cancer biology are necessary to effectively deal with the tumor heterogeneity that exists within and between patients. In the following we highlight a novel approach to develop a biological therapeutic strategy designed to overcome many of the challenges associated with the treatment of advanced metastatic disease. Oncolytic viruses (OVs) are tumor selective replicating therapeutics that have the potential to eliminate malignancies through both direct cell lysis and generation of an in situ anti-tumor immune response. Though conceptually, OVs appear to be an ideal cancer therapeutic, to date, limited clinical activity has dampened overall enthusiasm for the platform. However exciting clinical data over the last 12 months has buoyed the field. A trial led by Antonio Chiocca using an oncolytic herpes virus containing a transcriptionally regulated virulence gene, demonstrated exciting clinical data following locoregional delivery in glioma patients.^2^ A second glioma trial testing locoregional therapy using Adenovirus based oncolytic called DNX-2401 again provided some provocative survival benefit in select patients.^3^ Finally, recent data from the BOND-003 Phase III study testing of an oncolytic adenovirus expressing human GM-CSF (*Cretostimogene Grenadenorepvec*) locally delivered to non-muscle invasive bladder cancers demonstrated a 76% complete response rate.^4^ These clinical data are particularly exciting as the studies were carried out with “*1st generation*” OVs that were originally described 20+ years ago and only one of the platforms contains and expresses a therapeutic transgene! We argue that more sophisticated OV strategies that truly exploit both the oncolytic and cancer gene therapy aspects of the platform are likely to generate even more compelling clinical results. A recent paper from Yuan Ping’s research group published in Nature Biotechnology^1^ represents a combination of novel strategies that builds on the strengths and overcomes some of the limitations of the oncolytic virus platform (Fig.1). As discussed above, locoregional therapy using OVs has shown clinical responses however, the *“Holy Grail”* for the field, is to develop a technology that can facilitate intravenous delivery of OVs to sites of metastatic disease coupled with activation of a systemic anti-tumor immune response. Chen et al.^1^ designed an oncolytic adenovirus (OA) wherein a critical viral gene, E1A, is controlled by the cellular hTERT promoter thus restricting virus growth to telomerase overexpressing tumor cells. The authors then developed an interesting technology to cloak the OA using membranes from tumor cells or membranes engineered to display tumor antigens (e.g., peptide:MHC complexes or cell surface tumor markers). Using an extrusion process, the OA was enveloped with the cell membrane mimetic and then co-incubated with T cells, specific for the membrane-displayed antigen. Chen et al.^1^ demonstrate this can be accomplished either using tumor infiltrating lymphocytes (TILs) or alternatively T cells engineered to express a particular chimeric antigen receptor or CAR. This latter approach has the benefit of being able to use an “*off the shelf*” membrane preparation made from a single master cell bank engineered to express the appropriate antigen. In either case the strategy: (1) cloaks the oncolytic virus from detection by the innate and adaptive immune system; (2) tethers the OA to a mobile anti-cancer T cell to allow systemic carriage into the tumor bed and; (3) facilitates co-delivery of two therapeutics to the same population of cancer cells. This last characteristic may be particularly critical as the OA is also engineered to express guide RNAs and the Cas9 enzyme to facilitate disruption of the *PDL1* gene in infected tumor cells. An alternative approach would be to encode within the OV shRNAs or artificial microRNAs (amiRNAs) targeted against *PDL1* gene transcripts. It’s been demonstrated that OVs encoding amiRNAs not only down regulate target genes in infected cells but also adjacent uninfected cells through exosomal transfer of the amiRNA.^5^ Without a head to head comparison it is difficult to determine which approach (e.g., CRISPR vs. shRNA) is preferred however one can speculate that since the gene disruption strategy does not require sustained expression of the CRISPR enzyme it may be more effective at removing the immune checkpoint protein than an RNA targeting strategy.

Others have demonstrated that the use of cellular carriers can facilitate OV deposition into the tumor bed however the strategy used by Chen et al. is particularly interesting as it does not depend upon active infection and virus replication in the carrier cell. Indeed it may be that by engaging the T cell TCR through an MHC:peptide complex helps retain the cloaked virus on the cell surface till it is delivered to the target tumor cell. The use of the OA to carry out site directed deletion of PDL1 is an interesting application of OV directed cancer gene therapy. While others have shown that OVs can be engineered to express secreted antibodies to antagonize immune checkpoint inhibitors Chen et al. demonstrate that the localized deletion of the *PDL1* gene coupled with OA mediated oncolysis in the same vicinity as tumor targeted T cells appears to be sufficient to trigger a systemic, therapeutic immune response.

Thus like the multi-tooled Swiss Army Knife, Chen et al. have created a multi-functional therapeutic that deletes a critical immune checkpoint inhibitor gene in infected tumor cells, directly adjacent to tumor selective T cells and then in a feed forward process, lyses cancer cells releasing further tumor antigens to a locally activated immune system. Interestingly simply mixing the OA with a tumor selective T cell and then co-administering in vivo was less effective than the formulated “OA:T cell complex” demonstrating the importance of geographical co-delivery. Perhaps the multi-virus delivery by the T cell following systemic delivery coupled with localized immune activation simulates what is achieved by locoregional therapy?
